# Clinical and dermatoscopic features of temporal triangular alopecia in infants

**DOI:** 10.1111/srt.13294

**Published:** 2023-03-02

**Authors:** Zhiwei Guan, Weijie Shi, Min Ren, Tiantian Bi, Haihui Su

**Affiliations:** ^1^ Department of Dermatology Tianjin Children's Hospital Tianjin China; ^2^ Department of Dermatology Tianjin Xiqing Hospital Tianjin China

**Keywords:** dermatoscopy, infant, temporal triangular alopecia

## Abstract

**Objective:**

To summarize the clinical and dermatoscopic features of temporal triangular alopecia in infants and explore the clinical significance of dermatoscopy in the diagnosis of triangular alopecia temporalis in infants.

**Methods:**

A retrospective analysis was performed on 20 children with temporal triangular alopecia diagnosed in the dermatology clinic of Tianjin Children's Hospital from January 2015 to December 2021. Dermatoscopy was performed on all children, and images were collected.

**Results:**

The clinical features of 20 children were 15 males and five females, all of which were born immediately after birth; There were eight cases (40%) in the left temporal region, 10 cases (50%) in the right temporal region, one case (5%) in the head region, and one case (5%) in the occipital region; 19 cases were single (95%), one case was multiple (5%); There were 21 skin lesions, 15 triangular lesions (71.4%), four quasi‐circular lesions (19%), and two lance‐shaped lesions (9.5%). Trichoscopic features: The hair follicle opening in all skin lesions is normal, and the hair follicle opening can be seen with fluffy hair (vellus hair). The vellus hair is evenly distributed, and the length is diverse (both short and long vellus hair exist in the same hair loss area). There are 14 cases of white vellus hair (70%), five cases of white spots (25%), one case of honeycomb pigment pattern (5%), and one case of vascular dilation pattern (5%).

**Conclusion:**

Temporal triangular alopecia in infants has typical clinical and dermatoscopic characteristics, and the dermatoscopy can provide clinical basis for its diagnosis and differential diagnosis.

## 1 INTRODUCTION

Temporal triangular alopecia (TTA) also known as congenital triangular alopecia is a kind of asymptomatic permanent, localized, nonscar, noninflammatory alopecia. It is characterized by alopecia plaques without any potential skin changes, which are usually limited to the frontotemporal region of the scalp.^1^ The diagnosis is mainly based on its unique clinical manifestations. Due to the low incidence rate and insufficient understanding of the disease, it is easily confused with other types of localized alopecia clinically. The purpose of this study was to summarize the clinical and dermatoscopic features of TTA in infants and to evaluate the clinical significance of dermatoscopy assisted diagnosis of TTA.

## 2 MATERIALS AND METHODS

### 2.1 Patients

Twenty children with TTA, aged from 10 months to 4 years old, with an average age of 1.98 years, were collected from the outpatient department of dermatology of Tianjin Children's Hospital from January 2015 to December 2021.

### 2.2 Diagnostic criteria

(1) Triangular or lance‐shaped alopecia in the frontotemporal region of the scalp. (2) Dermatoscopic examination showed that normal hair follicle opening, with vellus hair, was surrounded by normal terminal hair. (3) Dermatoscopy showed no black spot sign, yellow spot sign, hair dystrophy, and reduction of hair follicle opening. (4) There was no obvious hair growth after the vellus hair was confirmed by dermatoscopy and clinical examination.^2^

### 2.3 Dermatoscopy

The Dermoscopy—II desktop dermatoscope of Beijing Demetricom was used to observe 21 skin lesions in 20 children with 20 times magnification. The lens is in parallel contact with the skin lesion, and 75% ethanol is used as the infiltrating liquid. Adjust the best angle and image definition, and collect the dermoscopic image of the skin lesion. Dermatoscopic images were collected by the same staff.

## 3 RESULTS

### 3.1 Clinical features and skin lesions

Fifteen males and five females, all of which were born after birth; There were eight cases (40%) in the left temporal region, 10 cases (50%) in the right temporal region, one case (5%) in the head region, and one case (5%) in the occipital region; 19 cases were single (95%), one case was multiple (5%). There were 21 skin lesions, 15 triangular lesions (71.4%), four quasi‐circular lesions (19%), and two lance‐shaped lesions (9.5%). The long axis is about 2–8 cm, and the average long axis is about 3.8 cm. A large amount of fine and soft vellus hair can be seen in the lesion area, which is difficult to remove. The boundary of the hair loss area is clear, and the scalp surface is normal, without erythema, scale and atrophy.

No other systemic diseases occurred in all cases. See Figure 1

FIGURE 1
(A–H) Left temporal part, (I‐Q) Right temporal part, (R) head restraint, (S) head top, (T) right temporal, two places, (B, M, O, and R) round; (T) triangle and lance‐shaped.

**Figure:**
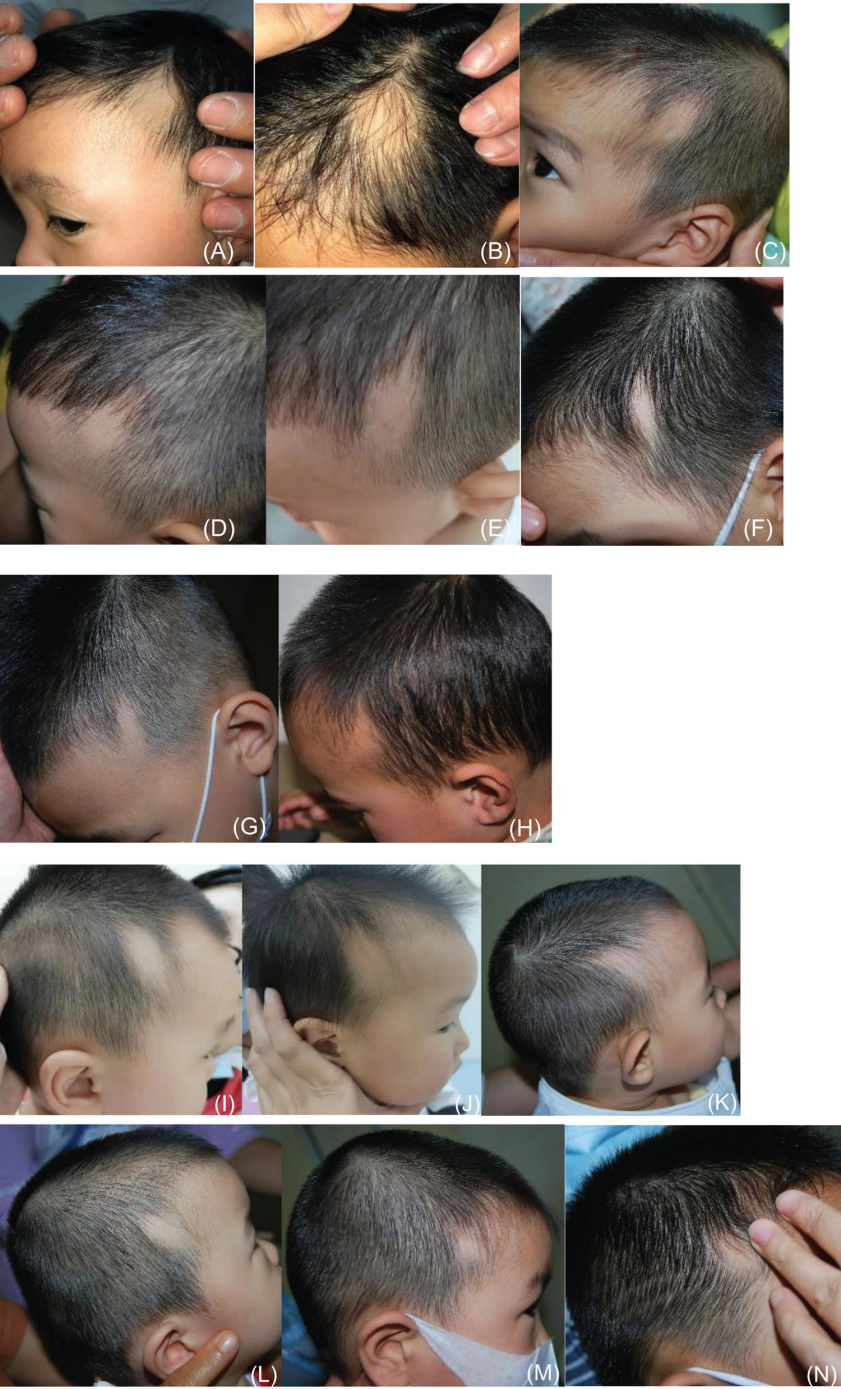

**Figure:**
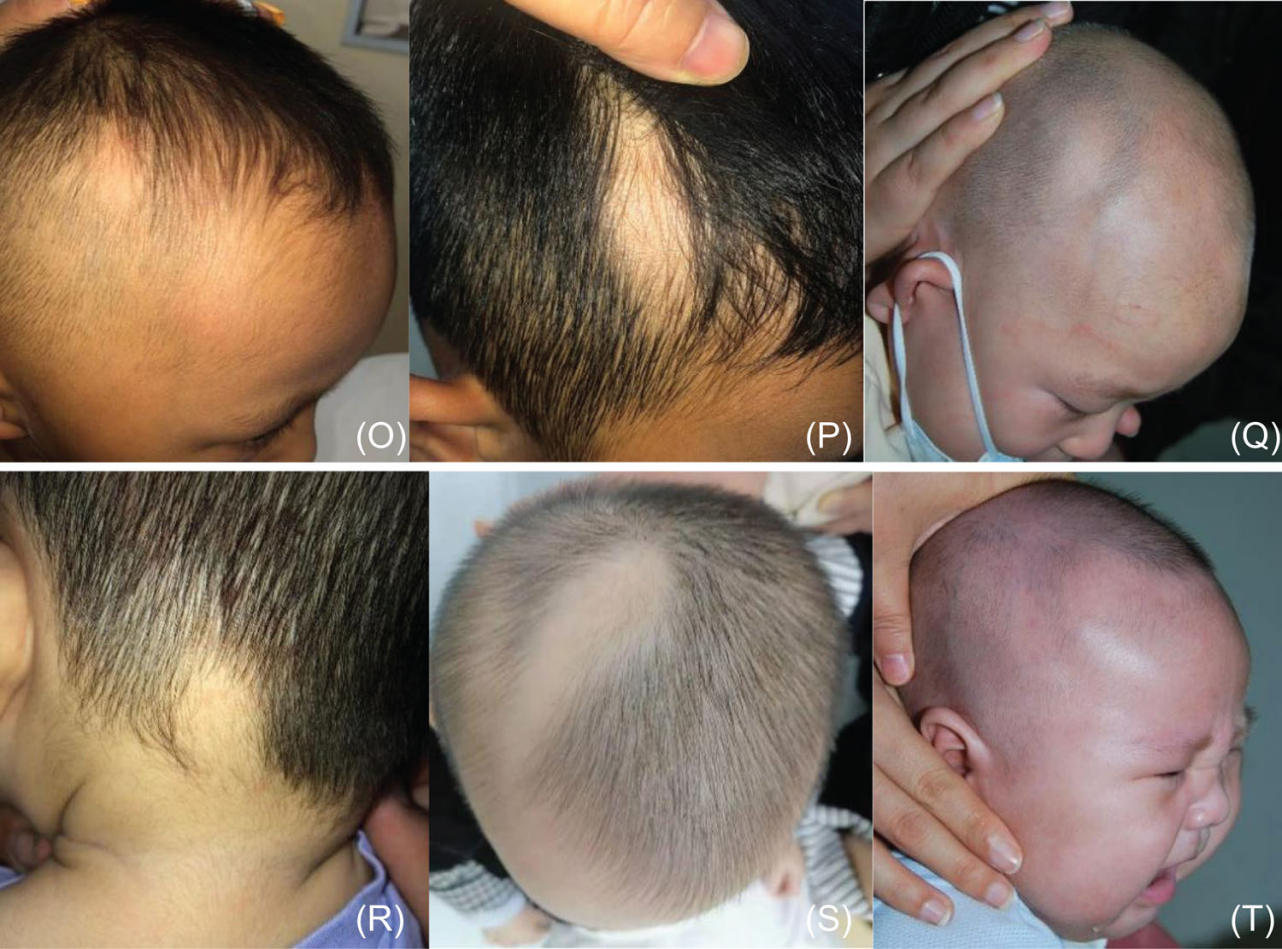

### 3.2 Dermatoscopic features

**FIGURE 2:**
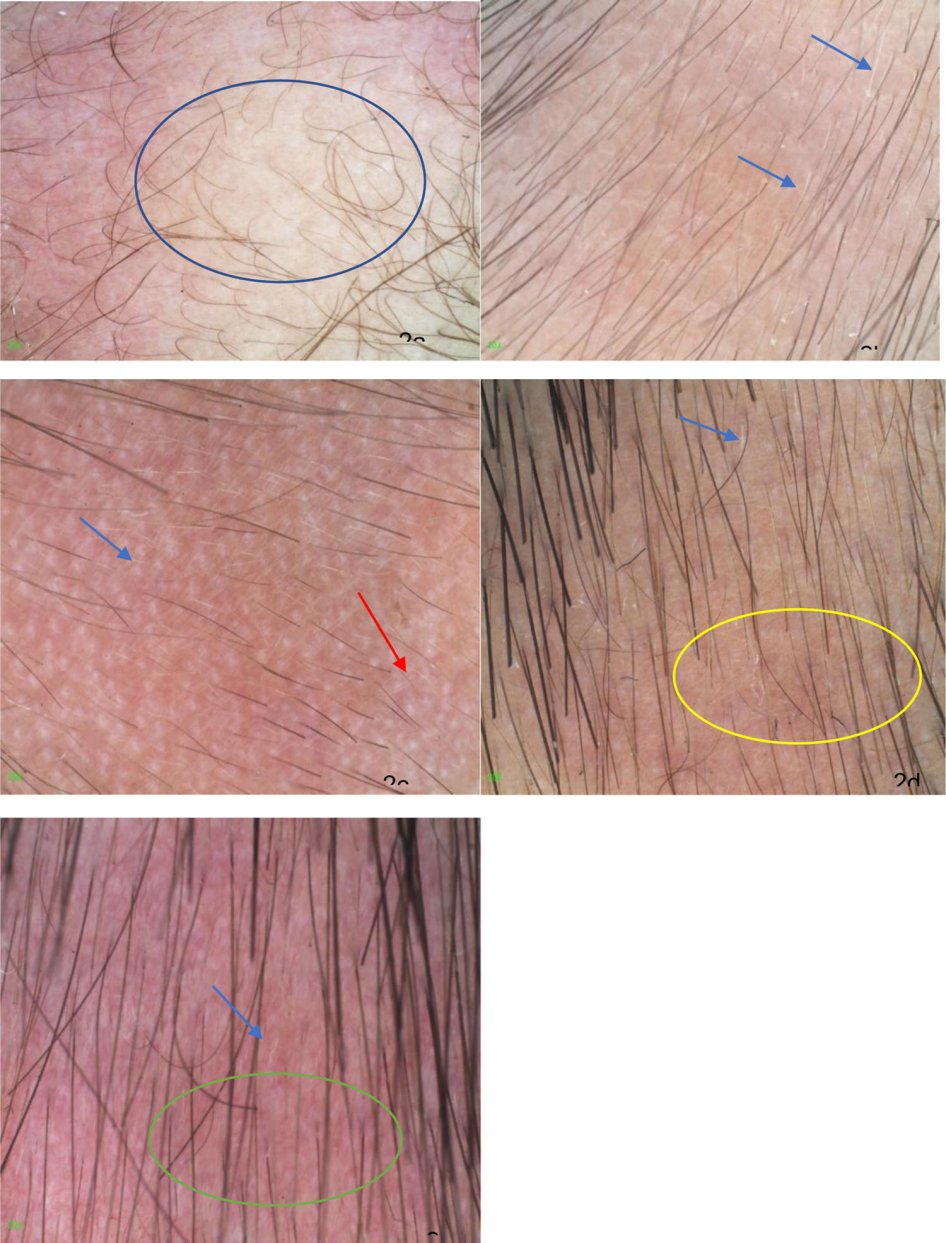
Dermatoscopic features (polarized light × 20), (A) vellus hair length diversity (blue circle), (B) white vellus hair (blue arrow), (C) white dot (red arrow), (D) honeycomb pigment pattern (yellow circle), (E) vascular dilation pattern (green circle).

All skin lesions have normal hair follicle openings, and there are plush hair (vellus hair) at the hair follicle opening. The vellus hair is surrounded by the terminal hair, and the vellus hair is evenly distributed, and the length is diverse (both short and long vellus hair exist in the same hair loss area). There were 14 cases of white vellus hair (70%), five cases of white spots (25%), one case of honeycomb pigment pattern (5%), and 1 case of vascular dilation pattern (5%). The skin color of all skin lesions is normal, without atrophy, nodule and ulceration. All skin lesions had no black spot sign, yellow spot sign, broken hair or exclamation mark hair, and the hair stretch test was negative (See Table 1; Figure 2). Both direct microscopic examination and Wood examination of fungi were negative.

**TABLE 1:** Dermatoscopic characteristics of patients with temporal triangular alopecia

Features	Number of patients
Vellus hair	20 (100%)
White vellus hair	14 (70%)
White spot	5 (25%)
Honeycomb Pigment Mode	1 (5%)
Vasodilation pattern	1 (5%)

## 4 DISCUSSION

TTA was first described by Sabouraud in 1905, and fewer than 200 cases have been reported so far.^3^ According to the literature, the incidence rate is only 0.11%,^4^ which is a rare disease. In recent years, TTA has been regarded as a relatively common disease with insufficient recognition and diagnosis. Only a few affected people can be diagnosed clearly when they seek treatment, and many children have been misdiagnosed.^5^ According to the literature,^6^ TTA mainly occurs in the frontotemporal region (more than 95%) and occipital region (2.5%), which is “triangular,” “oval,” “lancet,” or lance‐shaped,^7^ with unilateral occurrence accounting for 79%, bilateral occurrence accounting for 18.5%, and occipital region accounting for only 2.5%. The results of this study show that TTA can occur in both men and women, with a large proportion of men. The incidence rate of both sides was basically the same, with temporal involvement accounting for the highest proportion (90%), and head and occipital involvement accounting for 5%. Most of them are triangular (71.4%), quasi‐circular (19%) and lance‐shaped (9.5%). According to the literature, most TTAs appear at birth (36.5%) or 2–9 years old (58.8%)^8^ and remain unchanged thereafter (that is, they do not grow hair), without any symptoms. All children in this study are born. Considering that the data reported in the literature may be related to the fact that the child was only noticed and diagnosed by his family when he was about 1–2 years old.

Under the TTA skin microscope, all hair follicle openings have vellus hair, which is evenly distributed and of different length, reflecting the diversity of vellus hair length and surrounded by normal terminal hair. This pattern represents the typical characteristics of TTA.^9^ All the cases in this study conform to the above characteristics. White vellus hair can be seen in 75% of the cases. White vellus hair cannot be recognized by naked eyes but can be clearly observed through dermatoscope. Therefore, the white vellus hair that can only be recognized by dermatoscope is considered as a sensitive indicator of TTA. It is noteworthy that in this study, 13 children had a history of seeing a doctor outside the hospital and had been misdiagnosed as alopecia areata without the assistance of dermatoscopy only in clinical diagnosis. Five of them had received external steroid therapy for at least 1 month without any improvement, and the misdiagnosis rate was as high as 65%. The white spot in the skin mirror corresponds to the sweat gland or sebaceous gland structure on the scalp of the hair loss area, which appears in 25% of TTA children. Only one case of honeycomb pigment pattern appeared in the left temporal skin lesions of 4‐year‐old children, which may be related to the older children, darker skin color, long‐term sun exposure, and other factors. Only one case of vasodilation pattern appeared in multiple skin lesions of 10‐month‐old children, which may be related to the younger age and thin and tender skin of the children. The above features are not characteristic, and more cases need to be accumulated for summary and analysis.

The differential diagnosis related to TTA mainly includes congenital skin hypoplasia, sebaceous nevus, alopecia areata, trichotillomania, telogen alopecia, and tinea capitis. See Table 2 for details. All cases in this study are postnatal, and the most easily confused are congenital skin hypoplasia and sebaceous nevus. Congenital skin hypoplasia is clinically characterized by pink or red atrophic translucent surface, and sebaceous nevus is clinically characterized by yellow‐orange velvet‐like plaque. However, these classic features may disappear soon after birth. Under the dermoscope, the independently distributed and relatively consistent yellow‐red globular block structure unrelated to hair follicles is the manifestation of sebaceous nevus,^10^ while the translucent area without any skin appendage opening is the dermoscopic manifestation of congenital skin hypoplasia. Alopecia areata usually occurs suddenly and the day after tomorrow. There may be multiple alopecia areas with self‐limiting. Typical skin microscopic signs of alopecia areata are yellow spot sign, black spot sign, broken hair, exclamation mark hair and short vellus hair. The exclamation mark hair has diagnostic significance, and the yellow dot sign and short vellus hair are sensitive indicators. Black spot sign, exclamation mark, and broken hair are specific indicators. Here, it needs to be specially pointed out that dermatoscopy of TTA is characterized by the diversity of vellus hair length, while there is only short vellus hair (length < 10 mm) in alopecia areata.^11^ Trichotillomania is common in adolescents and rarely occurs in infants. It mainly occurs in the parietal and frontal lobes. Dermatoscopy typically shows black spot sign, broken hair, hair tip looks like a “broom” or “brush,” hair trunk stump is split and curled, and skin can see bleeding spots and pigmentation. Under the microscope, many hairless hair follicle openings can be seen in the hair loss skin during the rest period, and there are a lot of new hair.^12^ Dermatoscopic manifestations of tinea capitis are broken hair, fine scales, spiral, comma‐like hair, and dystrophic hair. The fungus was positive by microscopic examination.

**TABLE 2:** Dermatoscopic differential diagnosis of temporal triangular alopecia

Disease name	Dermatoscopic appearance
Triangular alopecia of temporal region	Vellus hair with varied length, white hair
Congenital skin hypoplasia	Translucent appearance, no skin appendages
Sebaceous nevus	Bright yellow dots, not related to hair follicles
alopecia areata	Yellow spot sign, black spot sign, broken hair, exclamation mark hair, short vellus hair
Trichotillomania	Black spot sign, broken hair, splitting and curling of hair stem stump
Telogen alopecia	Hair follicles with hairless trunks and new hair
Tinea capitis	Broken hair, scale, spiral hair, comma hair

TTA generally does not require treatment intervention. Hair transplantation and surgical resection are the main treatment options at present. Both topical and systemic steroid therapy are ineffective.^13^ Through this study, we hope that more medical workers will understand TTA. TTA is a disease that needs to be considered in the face of localized hair loss in infants. The histopathological examination of invasive skin lesions is very painful for children and difficult for their families to accept. Dermatoscopy is simple, rapid and non‐invasive. Dermatoscopy can indicate vellus hair and white vellus hair with length diversity that cannot be observed by naked eyes, providing strong evidence for the diagnosis of TTA, improving the diagnostic accuracy, reducing the misdiagnosis rate, and avoiding improper treatment.

## CONFLICT OF INTEREST STATEMENT

The authors have no conflict of interest to declare.

## Data Availability

No
